# Face processing and early event-related potentials: replications and novel findings

**DOI:** 10.3389/fnhum.2023.1268972

**Published:** 2023-10-25

**Authors:** Nicolas M. Brunet

**Affiliations:** Department of Psychology, California State University of San Bernardino, San Bernardino, CA, United States

**Keywords:** ERP, EEG, N170, P200, age, face inversion, face mask, amplitude

## Abstract

This research explores early Event-Related Potentials (ERPs) sensitivity to facial stimuli, investigating various facial features aimed to unveil underlying neural mechanisms. Two experiments, each involving 15 undergraduate students, utilized a multidimensional stimulus set incorporating race, gender, age, emotional expression, face masks, and stimulus orientation. Findings highlight significant modulations in N170 and P200 amplitudes and latencies for specific attributes, replicating prior research and revealing novel insights. Notably, age-related facial feature variations, facial inversion, and the presence of face masks significantly impact neural responses. Several speculative explanations are proposed to elucidate these results: First, the findings lend support to the idea that the increased N170 amplitude observed with facial inversion is closely tied to the activation of object-sensitive neurons. This is further bolstered by a similar amplitude increase noted when masks (effective objects) are added to faces. Second, the absence of an additional amplitude increase, when inverting face images with face masks suggests that neural populations may have reached a saturation point, limiting further enhancement. Third, the study reveals that the latency deficit in N170 induced by facial inversion is even more pronounced in the subsequent ERP component, the P200, indicating that face inversion may impact multiple stages of face processing. Lastly, the significant increase in P200 amplitude, typically associated with face typicality, for masked faces in this study aligns with previous research that demonstrated elevated P200 amplitudes for scrambled faces. This suggests that obscured faces may be processed as typical, potentially representing a default state in face processing.

## 1. Introduction

Electroencephalography (EEG) plays a crucial role in cognitive neuroscience, particularly in studying facial processing (Rossion, 2014). The favorable location of the Fusiform Face Area (FFA) in the inferior temporal cortex, specialized for facial stimuli, enhances EEG data collection through scalp sensors. Researchers utilize EEG to investigate FFA activity, exploring various factors and cognitive processes. Participants encounter a range of facial and non-facial stimuli, engaging in tasks like memory and identification. This approach yields event-related potentials (ERPs) obtained through EEG data averaging, revealing positive and negative voltage deflections (ERP components) (Rossion and Jacques, 2008). These components correspond to distinct neural sources, encapsulating different facial processing stages.

Key ERP components commonly addressed in early vision studies include the P1 (P100), a positive deflection occurring approximately 100 ms post-stimulus onset. It primarily reflects occipital lobe activity and exhibits heightened responses to facial stimuli (Itier and Taylor, 2004; Herrmann et al., 2005; Kaltwasser et al., 2014; Moradi et al., 2017; Gantiva et al., 2020). The N170, a negative deflection within the 130–200 ms timeframe, originates from FFA, showing larger amplitudes for faces and a pronounced sensitivity to facial features and configurations (Johnson, 2005; Rossion and Jacques, 2011; Hinojosa et al., 2015; Schindler and Bublatzky, 2020). Lastly, the P2 (P200) component peaks around 150–275 ms post-stimulus onset, characterized by its sensitivity to attentional processes (Carretié et al., 2013) and facial prototypicality (Schweinberger and Neumann, 2016).

Studying the Fusiform Face Area (FFA) is of utmost importance because it sheds light on a fundamental aspect of brain processing: the brain’s specialization in processing specific information categories like faces, places, tools, and body parts, which likely extends to other sensory modalities and cognitive processes (Pascual-Leone and Hamilton, 2001).

For a visual stimulus to elicit a face-sensitive N170 response, it must contain enough information in terms of local elements and their arrangement to create the perception of a face (Rossion and Jacques, 2011). Due to the cyclical resurgence of respiratory viruses, face masks are expected to persist. A recent study (Freud et al., 2022) reports that adding masks significantly impairs face recognition, contradicting the notion of easy adaptation to a masked world. This raises the question of how mask-wearing affects early face processing and the retrieval of vital facial information like age, gender, race/ethnicity, and facial expressions, characteristics, usually easily discernible in unmasked faces.

To that extent, a set of facial stimuli was carefully curated, varying across five binary dimensions: gender (male/female), race (white/black), facial expression (happy/angry), age (young/old), and the presence or absence of a face mask. This approach not only facilitates the exploration of interactions among these variables but also offers the opportunity to reexamine prior research with divergent findings regarding the influence of these factors on P100 and N170 processes. Some studies, for instance, reported no discrimination by N170 based on emotional expression (Eimer and Holmes, 2002; Herrmann et al., 2002; Eimer et al., 2003), while others noted larger amplitudes in response to fearful faces (Batty and Taylor, 2003; Williams et al., 2006; Blau et al., 2007; Luo et al., 2010), some even at earlier latencies (Walker et al., 2008). Social category modulation of N170 has also produced mixed results, with some studies showing no effect (Caldara et al., 2004; He et al., 2009; Wiese et al., 2009) or increased N170 responses to other-race faces (Walker et al., 2008). Conversely, no gender effects have been reported on P100 or N170 components (Mouchetant-Rostaing et al., 2000; Mouchetant-Rostaing and Giard, 2003). Regarding facial age, both young and older participants exhibit heightened N170 amplitudes when presented with older faces compared to younger ones (Wiese et al., 2008; Wiese, 2012). However, the N170’s sensitivity to age-related factors diminishes when age and race/ethnicity factors are presented concurrently, suggesting potential modulation by contextual or task-related variables (Wiese, 2012). These findings indicate that N170 responsiveness to age differs from its reactivity to race/ethnicity. These discrepancies across studies likely stem from variations in stimulus characteristics, task demands, experimental design, and stimulus presentation, posing challenges for direct comparisons.

**Figure 1:**
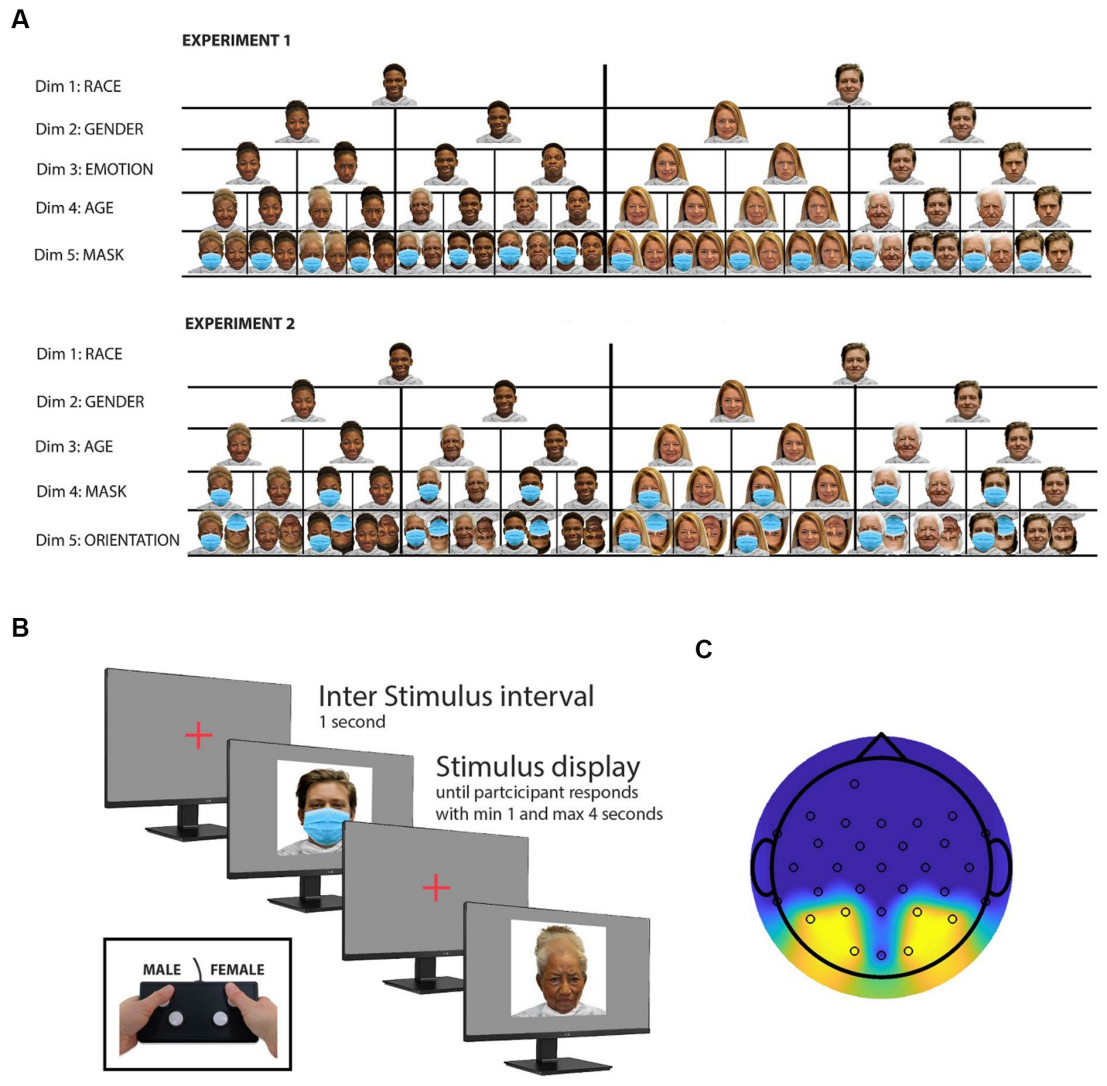
Experimental procedures and stimulus selection. **(A)** Illustration of experimental stimuli. This panel presents representative samples of the stimuli employed in two distinct experiments, each comprising a total of 416 stimuli. These stimuli were strategically chosen to investigate alterations in neural responses to facial stimuli, specifically examining the impact of face masks. The stimulus set was meticulously designed to encompass a balanced representation of various facial attributes, including an equitable distribution of both Caucasian and African American faces (dimension 1), male and female faces (dimension 2), as well as happy and angry facial expressions (dimension 3), drawn from the openly accessible RADIATE face database. To introduce an additional dimension, an “older” rendition of the faces was created using the photo editing application FaceApp. Furthermore, a fifth dimension (presence or absence of a mask) was introduced by digitally incorporating masks onto every image using Adobe Photoshop. For enhanced interpretability of the study results, a secondary experimental study was devised, which also featured inverted face images. Notably, the “emotion” dimension was excluded from the stimulus set in this second study to prevent an unwieldy number of trials. **(B)** Stimulus presentation and response task. All 416 stimuli are presented in random order. Participants are instructed to use a button box to indicate whether the displayed face is male or female. Faces are displayed for a minimum of 1 s and remain on screen until a participant response is recorded or up to 4 s if no response is detected. The intertrial interval lasts 1 s, during which a cross is displayed in the center of the monitor. **(C)** Topographic map with sensor locations. This panel illustrates the topographic map displaying the sensor locations utilized in both experiments. The reported study results are based on the averaged signal recorded from the sensors highlighted in yellow. However, topographic maps based on all sensors can be found in the Supplementary material section (see Supplementary Figures S1–S5).

The findings from 15 participants in the study revealed a significant and substantial increase in both N170 and P200 activity when comparing masked and unmasked faces, as elaborated in the Results and Discussion sections. This heightened N170 response bears a resemblance to the pattern observed with inverted faces (Rossion et al., 2000), which is thought to involve the additional engagement of object-sensitive neurons, supported by evidence from fMRI studies. These studies suggest that inverted faces become more akin to objects, eliciting stronger responses in object-sensitive brain regions (Yovel and Kanwisher, 2005; Epstein et al., 2006) while reducing activity in face-selective areas (Yovel and Kanwisher, 2005; Mazard et al., 2006). Given that face masks, perceptually, introduce an element of both object addition and reduced facial visibility, it is plausible that the hypothesis regarding inverted faces may extend to masked faces. Consequently, it can be hypothesized that inverted and masked faces elicit comparable ERP patterns. To investigate this hypothesis, the original experiment was modified by introducing a new binary dimension: stimulus orientation (upright/inverted). To maintain an equivalent number of trials as the original experiment, the binary dimension of emotional expression was simplified to include only “happy” faces (see Figure 1A). In this second experiment, an additional 15 participants were recruited.

To tackle the issue of the multiple comparison problem, a concern that frequently impacts ERP-related research and has been thoroughly discussed by Luck and Gaspelin (2017), the analysis focused on a single electrode combination for all assessments and a robust non-parametric randomization test to evaluate variations in amplitude and latencies among the various conditions (see Analysis in the Materials and methods section for more information).

## 2. Materials and methods

### 2.1. Subjects

This study involved two experiments, each with 15 undergraduate students. In experiment 1, there were 10 female participants and 5 male participants, all with a mean age of 24 years. Experiment 2 consisted of 12 female participants and 3 male participants, with a mean age of 25 years. All participants were psychology majors at the California State University of San Bernardino, and they received class credit in appreciation of their participation. Prior to their involvement, each student provided informed consent, and no student participated in more than one experiment.

### 2.2. Stimuli

To generate the image dataset used for Experiment 1, original face images from 26 white and 26 black models (equally distributed across gender) were selected from the RADIATE database (Conley et al., 2018). This database provides open-access face stimuli, featuring racially and ethnically diverse models displaying various emotional expressions. Two emotional expressions, namely “happy” and “angry,” were chosen for each model, resulting in a set of 104 unique face images. To expand the dataset, an AI aging filter (FaceApp) was employed to create an “older version” of each model, effectively doubling the number of stimuli. Subsequently, the dataset was doubled again by digitally adding a facemask to each face image, employing Adobe Photoshop. As a result, the dataset was expanded to include a total of 416 face images. For Experiment 2, the initial image dataset from Experiment 1 was halved by removing all “angry” faces. Then, the dataset was brought back to a total of 416 images by adding an inversed version of the remaining faces. Throughout both experiments, all face images were thus equally divided based on several attributes, including race (black/white), gender (male/female), age (young/old), use of face mask (mask/no mask), and emotional expression (happy/angry). In Experiment 2, an additional attribute, face orientation (upright/inverted), was considered, in place of emotional expression. Figure 1A illustrates examples of the stimuli used for experiments 1 and 2, featuring one black and one white male and female model and the stimuli derived from it.

### 2.3. Experimental procedure and EEG equipment

The experiment involved participants wearing a 64-channel EEG-cap (BrainVision), with only 32 channels effectively utilized (sampling rate of 500 Hz) and the reference electrode placed at the FCz location, following the standard 10–20 EEG system. The EEG electrodes were connected to a BrainVision actiCHamp active channel amplifier (BrainVision) and checked for proper conductivity (impedance below 5kΩ for each electrode) before starting the recording. During the recording session, participants were seated in a dimly lit, quiet room, in front of a 19-inch Dell monitor, positioned 50 cm away from their heads. The experiment presented 416 face images (see “Stimuli”) sequentially, each displayed at the center of the screen with a visual angle of 17° × 23°. The order of the images was randomized to minimize any potential biases. To ensure participants’ attention, they were instructed to indicate the gender of each face using a button box (see Figure 1B). Each face was displayed for at least 1 s, and disappeared from the screen as soon as it was evaluated, followed by a 1-s inter-trial interval displaying a cross at the center of the screen before the next face appeared (see Figure 1B). Participants were encouraged to respond as quickly and accurately as possible. Any images not evaluated within 4 s disappeared from the screen to maintain the experimental flow. The entire experimental paradigm was created using Experiment Builder by SR Research.

### 2.4. Analysis

#### 2.4.1. Stimulus presentation and data acquisition

Each stimulus used in both Experiment 1 and 2 contained a small black square engineered in the bottom right corner, allowing precise timing information through a screen-positioned photodiode. This setup ensured accurate timestamps for the onset and offset of the 416 face stimuli used in the experiments.

#### 2.4.2. Data segmentation and preprocessing

The EEG data were analyzed offline using the FieldTrip Matlab software toolbox (Oostenveld et al., 2011). Noisy trials were removed using the Fieldtrip data browser function, and the timestamps were utilized to generate 416 data segments, each lasting 2 s, spanning 0.5 s before stimulus onset to 1.5 s after onset. This broader range than needed was selected to prevent edge effects caused by preprocessing. Subsequently, the raw data was filtered (3–45 Hz) and demeaned. The unusual high-pass filter (3 Hz) was selected because it effectively removed slow drifts observed in the EEG signal (Pinto et al., 2019). DFT notch filters were applied at 60 and 120 Hz.

#### 2.4.3. Electrode selection and grand average ERP waveforms

**Figure 2:**
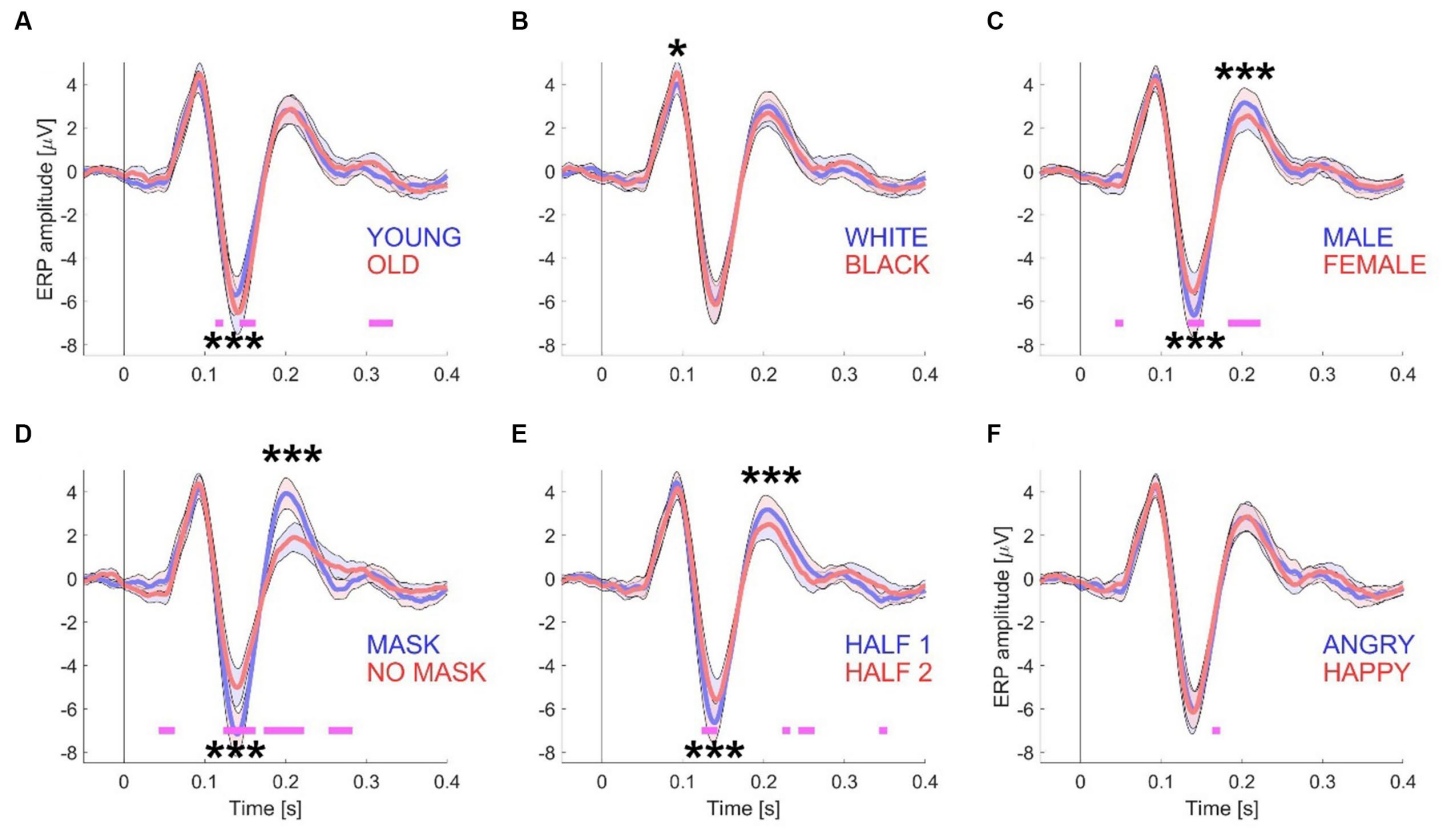
ERP waveforms for Experiment 1. This figure presents the grand mean ERP waveforms, computed from a total of 15 datasets with 416 trials. The waveforms are grouped according to specific conditions: “young/old” **(A)**, “white/black” **(B)**, “male/female” **(C)**, “mask/no mask” **(D)**, the first and second half of the session **(E)**, and “angry/happy” **(F)**. To generate the conditions depicted in each panel, the electrode data from the left-side cluster (P7, P3, and O1) and the right-side cluster (P4, P8, and O2) were first averaged across all participants. The shaded areas in the graphs represent the standard error (+/−) with *N* = 15 participants. Significance testing along the waveforms for differences between the two conditions was conducted using paired *t*-tests (see Materials and methods) with an alpha level of 0.01 to account for multiple comparisons. Statistically significant differences are indicated by pink horizontal lines beneath the waveforms. For amplitude comparisons between the P100, N170, and P200 components of both conditions, a non-parametric randomization test was employed (see Materials and methods and Table 1). Statistically significant differences are represented by one (*p* < 0.05), two (*p* < 0.01), or three (*p* < 0.001) asterisks at the relevant locations.

**Figure 3:**
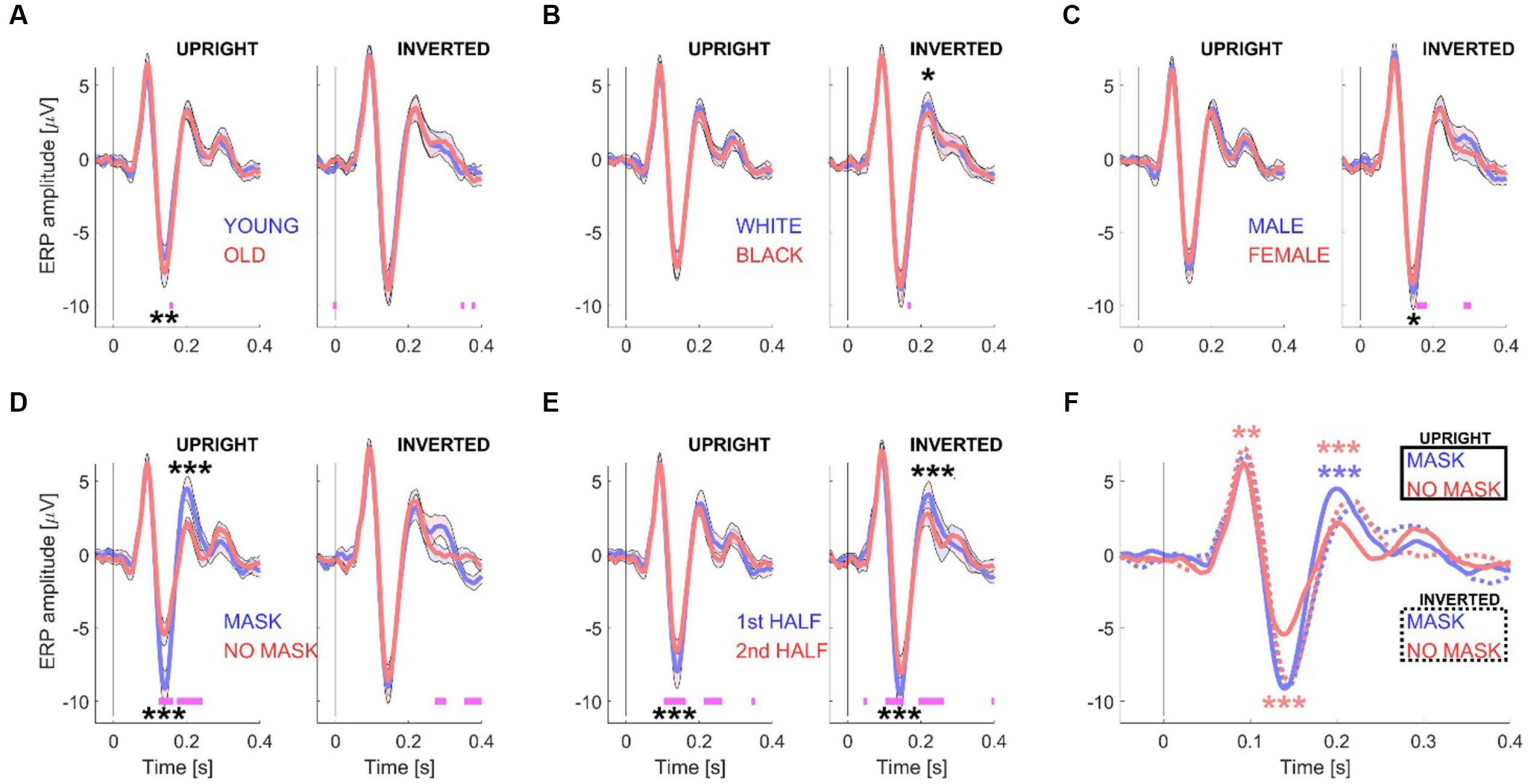
ERP waveforms for Experiment 2. This figure illustrates the grand mean ERP waveforms, derived from 15 datasets with 416 trials. Like Figure 2, the waveforms are grouped according to specific conditions: “young/old” **(A)**, “white/black” **(B)**, “male/female” **(C)**, “mask/no mask” **(D)**, and the first and second half of the session **(E)**. However, in Experiment 2, the analysis was performed separately for upright (left subpanels) and inverted (right subpanels) stimuli. **(F)** Displays the grand mean ERP waveforms for the “mask vs. no mask” conditions, both for upright stimuli (solid curves) and inverted stimuli (dotted curves). The shaded areas in the graphs represent the standard error (+/−) with *N* = 15 participants. Significance testing along the waveforms for differences between the two conditions **(A–E)** was conducted using paired *t*-tests (see Materials and methods) with an alpha level of 0.01 to account for multiple comparisons. Statistically significant differences are indicated by pink horizontal lines beneath the waveforms. For amplitude comparisons between the P100, N170, and P200 components of each pair of conditions **(A–E)** and for the two “mask” conditions or the two “no mask” conditions **(F)**, a non-parametric randomization test was employed (see Materials and methods and Table 1). Statistically significant differences are represented by one (*p* < 0.05), two (*p* < 0.01), or three (*p* < 0.001) asterisks at the relevant locations. The asterisks in panel F are color-coded to distinguish between comparisons of the two “mask” conditions (blue) and the two “no mask” conditions (red).

In alignment with established practices in the field (e.g., Gao et al., 2009), ERP waveforms and their associated components were computed by averaging across specific occipito-temporal electrodes, namely P7, P3, O1, O2, P4, and P8 (as illustrated in Figure 1C, highlighted in yellow). A consistent electrode selection was maintained across all ERP components to prevent multiple implicit comparisons (Luck and Gaspelin, 2017). However, data from all recorded sites were utilized to generate topographic maps, which can be found in the Supplementary material. Following the averaging of data for each pair of experimental conditions and each participant, grand average ERP waveforms were computed by averaging across the results from all 15 participants for each experiment. The shaded regions in Figures 2, 3 represent the standard error of the grand average, denoted as ±SE. To ensure smooth curves and error bands, a 5-point moving average with a window of 0.010 s was applied.

#### 2.4.4. Statistical analysis

To evaluate statistical distinctions along the ERP waveforms across experimental conditions, the study implemented a “running *p*-value” approach utilizing a paired *t*-test with a sample size of *N* = 15. This method involved assessing variations in intervals of 0.010 s (equivalent to 5 samples) throughout the ERP waveforms. Pink horizontal line segments were incorporated to indicate locations where *p*-values < 0.01 were observed beneath the curves for enhanced visual representation.

Although the study is structured around a factorial design featuring 5 independent variables, only three-way ANOVA (using the Matlab function anovan.m) were utilized to investigate interactions between different pairs of independent variables concerning the amplitudes of P100, N170, and P200 (see Supplementary material for results). This choice was made because higher-dimensional ANOVAs, would of have required averaging smaller trial quantities and potentially led to unstable ERP waveforms and associated components.

For the presentation of topographic maps illustrating the amplitudes of the various ERP components, the Matlab function plottopography.m was employed (available through the Mathworks file exchange). Detailed explanations regarding the derivation of ERP component peak values, utilized for both the ANOVA and the creation of topographic maps, are provided in the subsequent section.

#### 2.4.5. Non-parametric approach for amplitude comparison

Amplitude differences in the EEG components (P100, N170, P200) between two experimental conditions were explored using a non-parametric approach. This approach involved 100,000 iterations, during which—for each iteration—data from 416 trials for each participant were randomly divided into two pseudo conditions, yielding grand average ERP waveforms for each condition by averaging across occipito-temporal electrodes and participants (see above). Amplitude differences for each pair of pseudo-ERP waveforms were calculated within specific time windows: 80–120 ms (P100), 150–300 ms (P200), and 100–200 ms (N170) for each iteration. The resulting 100,000 amplitude differences were sorted from high to low, allowing computation of the ranking of the experimentally observed differences for each pair of experimental conditions and each ERP component of interest among the randomizations. For instance, a rank of 5 indicated that only 4 out of 100,000 randomizations yielded a greater amplitude difference, resulting in a probability of 5/100,000 (thus, *p* = 0.00005). In Experiment 2, this non-parametric test was performed separately for trials featuring upright and inverse face images, as well as for trials featuring face images with and without masks (see Table 1). An identical approach, using the same time windows for each ERP component, was also used to compute differences in latency between masked and unmasked faces, and upright and inverted faces.

**Table 1:** Non-parametric randomization test results.

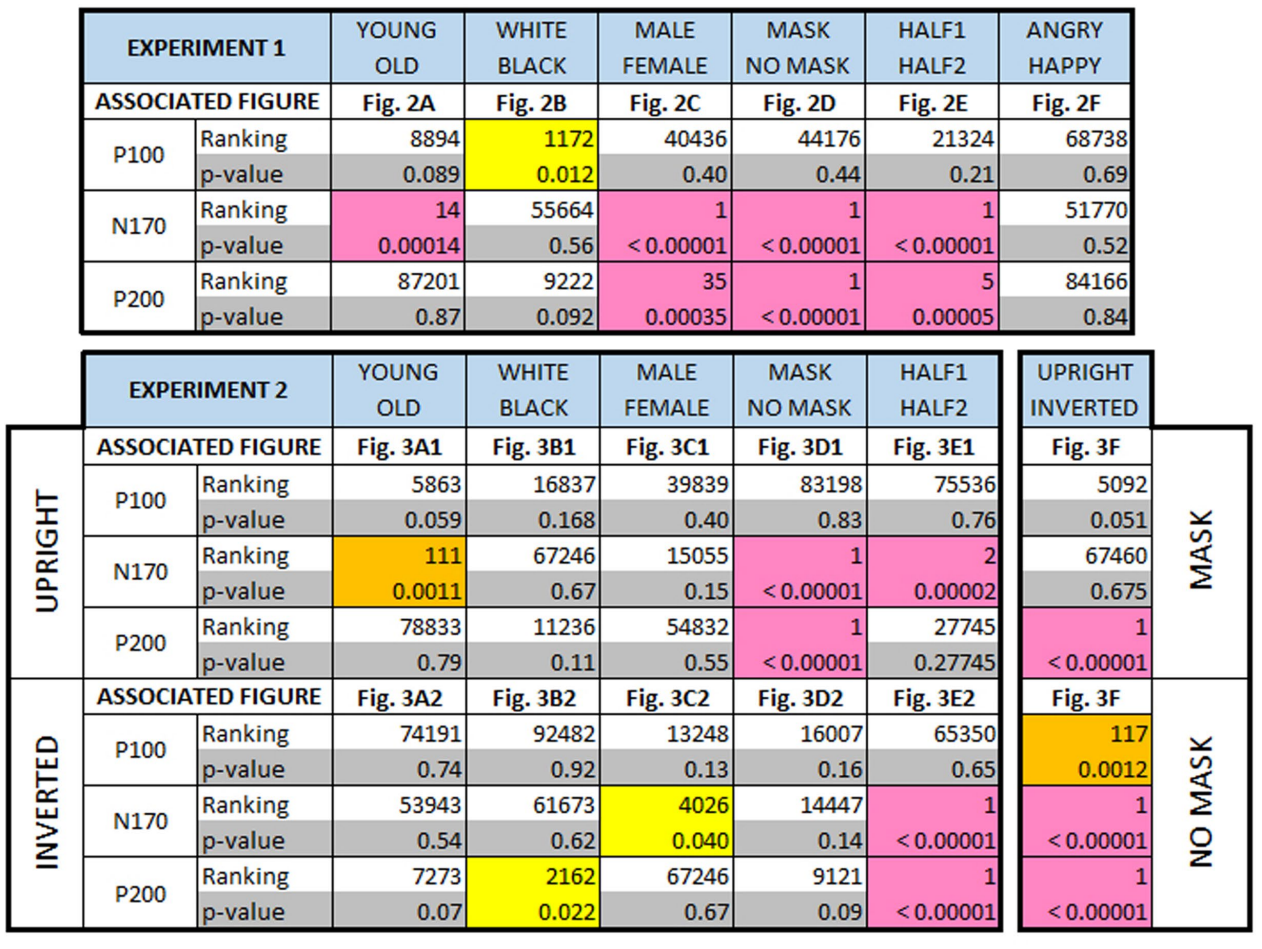

A non-parametric test (see Materials and methods for details) was conducted with 100,000 iterations to investigate potential differences in the amplitudes of key ERP components (P100, N170, and P200) among various conditions. Each iteration involved dividing all trials of all participants into two pseudo conditions, resulting in 100,000 amplitude differences for each ERP component. These differences were then ranked from highest to lowest, allowing for an assessment of their positions compared to those generated by randomizations. A rank of 1 indicated that none of the 100,000 randomizations resulted in a larger amplitude difference than the one measured. Based on these rankings, *p*-values were assigned. The table displays the rankings and *p*-values for each pair of conditions (as listed in the light blue shaded table header). The top part of the table presents the results obtained for experiment 1, while the detached bottom part shows the results obtained for experiment 2. In experiment 2, separate analyses were performed for upright and inverted face stimuli, with the exception of the statistics provided in the (detached) last column, where the analysis was conducted separately for face stimuli with and without a mask. Each comparison in the table corresponds to a specific figure panel (figure references are provided). To enhance clarity, a color code we used: gray shading for *p*-values above 0.05, yellow for *p*-values smaller than 0.05 but larger than 0.01, orange for *p*-values smaller than 0.01 but larger than 0.001, and pink for *p*-values equal to or lower than 0.001. The direction (left/right) of significant *p*-values, although not explicitly reported in the table, can easily be inferred by consulting the associated figure panels.

## 3. Results

### 3.1. Experiment 1

Participants viewed 416 images, which were presented one by one in random order. Their sole task was to identify whether each face image depicted a female or a male by using a button box (see Figure 1B).

The 416-image stimulus set was purposely designed to be split into two sets of 208 images each, based on five distinct face attributes (see Figure 1A for examples). Consequently, this division resulted in five pairs of grand averaged ERP waveforms. These pairs were categorized as follows: “young” and “old” (see Figure 2A), “white Caucasian” and “Black African American” (see Figure 2B), “male” and “female” (see Figure 2C), “mask” and “no mask” (see Figure 2D), and “angry” and “happy” (see Figure 2F). Additionally, for each participant, the trials were divided into two groups: those presented first (trials 1 to 208) and those presented last (trials 209–416) (see Figure 2E).

To visualize and analyze the differences in the measured EEG signals between the selected pairs of conditions (see Materials and methods), the following features were included in each panel: a shaded error band representing ± standard error for each waveform, short horizontal lines under each pair of waveforms to indicate the timepoints at which the waveforms statistically differed from each other (with alpha = 0.01), and asterisks (see caption Figure 2) to indicate differences in amplitudes between the waveforms for the key ERP components (P100, N170, and P200) that are relevant to this study, based upon a non-parametric test (see Materials and methods).

Results, obtained through a 3-way ANOVA, aimed at exploring interactions among the binary dimensions of “age,” “emotion,” and “mask,” as well as “gender,” “race,” and “mask,” and their impact on the amplitudes of the P100, N170, and P200 components, are presented in Supplementary Tables S1, S2. Complementing these findings, Supplementary Figures S1, S2 show topographic maps depicting amplitude distributions for these components under various conditions.

### 3.2. Experiment 2

Following experiment 1, a group of 15 new participants was recruited to replicate the study, incorporating notable modifications. Specifically, the “emotional expression” factor was omitted, and a new “stimulus orientation dimension” was introduced. These adjustments were informed by the findings of experiment 1, which highlighted substantial differences, particularly in N170 and P200 amplitudes, between masked and unmasked faces. In contrast, no discernible distinctions were observed between “angry” and “happy” faces. This alignment with the concept that emotional effects on early vision may not necessarily signify an influence of cognitive processes (Raftopoulos, 2023). Because similar amplitude differences have been reported for inverted faces (Rossion et al., 2000), the introduction of face stimulus orientation provided an opportunity to compare and study the effect of both modulations, and hence learn more about the underlaying neural mechanisms. For examples of stimuli used for Experiment 2, and how they differ compared with Experiment 1, see Figure 1A.

Similar to the results shown for Experiment 1 (see Figure 2), the panels in Figures 3A–E illustrates the results obtained from splitting the data based on one pair of experimental conditions. To examine the effect of inverting the stimuli, upright (left subpanels) and inverted (right subpanels) stimuli were analyzed separately. Additionally, to compare the effects of both masks and stimulus orientation, the data was split into four groups: no masks and masks, either upright or inverted (see Figure 3F).

The results, derived from a 3-way ANOVA, were directed toward investigating interactions among the binary dimensions of “stimulus orientation” and “mask,” along with either “age” (Supplementary Table S3), “gender” (Supplementary Table S4), or “race” (Supplementary Table S5), and their influence on the amplitudes of the P100 and N170 components (see Supplementary material). To complement these findings, Supplementary Figures S3–S5 present topographic maps illustrating amplitude distributions for these components under conditions involving masked vs. unmasked stimuli and upright vs. inverted faces.

### 3.3. Amplitude and latencies of the P100, N170, and P200

Table 1 provides a comprehensive summary of amplitude differences for all ERP components considered in both experiment 1 and experiment 2. A non-parametric randomization test was employed to compare amplitudes between any two experimental conditions (see Materials and methods). Additionally, this test helped identify latency shifts (not shown in Table 1) between different conditions for different ERP components. When analyzing the EEG response separately for inverted or upright face image stimuli, no latency shift was observed between masked and unmasked images. However, the response to inverted unmasked faces showed a significant delay compared to upright unmasked faces for the N170 (6 ms, *p* = 0.017) and the P200 (20 ms, *p* < 0.00001). Inverting masked faces also exhibited a delay, albeit not statistically significant (4 ms, *p* = 0.08), but a significant delay for the P200 (20 ms, *p* < 0.00001) compared to upright masked faces. For a more detailed interpretation of these findings, please see the “Discussion” section below.

## 4. Discussion

### 4.1. Factors influencing the N170 amplitude and latencies

This study found that N170 amplitudes increased with appearent complexity of processing faces. Older faces, for instance, known to be more challenging in terms of emotional expression recognition (Grondhuis et al., 2021), elicited larger N170 responses, noted in both Experiment 1 and 2, consistent with prior research (Wiese et al., 2008). Interestingly, the inversion of faces abolished this age-related difference (see Figure 3), although a separate study observed larger inversion effects for young faces compared to old faces (Wiese et al., 2008). The presence of a face mask, which also imposes additional processing demands, was also found to increase N170 amplitudes and P200 responses in both experiments, aligning with recent studies reporting similar N170 increments due to face masks (Prete et al., 2022; Proverbio and Cerri, 2023). It’s noteworthy, however, that another study (Żochowska et al., 2022) did not observe changes in N170 amplitudes with face masks. Remarkably, none of these studies noted a corresponding increase in P200 amplitude, possibly indicative of cognitive task variations.

Another critical aspect impacting face processing is facial inversion, a phenomenon evident across all conditions in our study except for the “masked face condition” (see below). This effect resulting in larger N170s is well-documented (Rossion et al., 2000; Rousselet et al., 2004; Sadeh and Yovel, 2010), been observed to impact the N250 (Hashemi et al., 2019; Abreu et al., 2023), an ERP component that falls outside the scope of this study. The absence of familiarity with faces (Ito and Urland, 2003), giving rise to the “own race bias effect,” has also been linked to heightened challenges in processing faces, resulting in larger N170 responses to other-race faces (Sun et al., 2014; Yao and Zhao, 2019). However, it’s important to note that the current study did not identify any sensitivity of early ERP components to race or skin color. This absence of sensitivity can be attributed to the predominant representation of Latino participants in the sample, which may not offer the requisite diversity to thoroughly investigate own-race effects. Furthermore, gender-based differences may affect N170 amplitudes, as evidenced by larger N170s for male faces, primarily due to the gender imbalance in our participant pool. Nevertheless, this finding warrants further investigation with a more balanced participant pool.

### 4.2. Potential neural mechanisms for increased N170 response to inverted faces

One plausible explanation for the enhanced N170 response to inverted faces lies in the early recruitment of additional neural mechanisms, rather than a simple increase in activity within existing neural populations during the N170 time-window. These findings align with Rossion’s hypothesis, which posits the involvement of object-sensitive neurons in augmenting the N170 amplitude observed for inverted faces (Rossion et al., 2000). This hypothesis may also be extended to elucidate the increased N170 response observed in our study for masked faces, as masks themselves can be considered objects. Intriguingly, the study demonstrates that the addition of face masks has a comparable effect on N170 amplitude as inverting the maskless face stimulus. However, combining a mask and inversion did not lead to an additive increase, suggesting a potential neural saturation point.

### 4.3. Delayed N170 response for inverted faces

The delay in the N170 response to inverted faces is often linked to alterations in the spatial relationships among facial features. Additionally, an amplification of the N170 component and a corresponding shift in latency have been associated with a reduced ability to recognize faces. For instance, a study demonstrated that gradually rotating facial images from an upright to an upside-down position resulted in a declining ability to identify faces (Jacques and Rossion, 2007). However, it’s important to emphasize that the reduced face recognition ability alone may not completely explain the observed delay in both the N170 and P200 components. This becomes evident in the present study, where no such delay was observed for faces with face masks compared to unmasked faces, despite face masks also significantly impeding face identification (Freud et al., 2022).

### 4.4. Factors influencing the P200 amplitude and latencies

The P200 component is well-established as being linked to configural face encoding, with more typical faces consistently yielding larger P200 amplitudes (Wuttke and Schweinberger, 2019). Conversely, as deviations from the norm increase, relatively smaller P200 amplitudes are typically observed (Halit et al., 2000; Latinus and Taylor, 2006; Schweinberger and Neumann, 2016). An intriguing revelation from our study is that both masked faces, as observed in our current investigation, and scrambled faces (Latinus and Taylor, 2006), elicit substantial P200 amplitudes. In contrast, a similar robust increase in the P200 component was not observed in response to facial inversion. This intriguing finding suggests that faces lacking spatial and configurational information may be processed as highly typical or in a default state, with the amplitude modulation being influenced by the presence of spatial face information.

Moreover, the inversion of faces was found to induce delays in both N170 and P200 latencies. This phenomenon has been noted previously (Latinus and Taylor, 2006), as well as in the case of Mooney faces compared to other face types. However, it is crucial to highlight that, unlike in our current study, previous research primarily emphasized the delay in N170 latency, as indicated by N170-to-P200 peak analyses. In contrast, this study revealed that the observed latency for inverted faces increased from 4–6 ms (N170) to approximately 20 ms (P200), demonstrating that this delay is not limited to one specific processing stage.

### 4.5. Effects of neural adaptation on the N170 and P200 components

The investigation of diminished ERP components resulting from repetition effects typically involves comparing responses to two identical faces or faces sharing common attributes (e.g., identity) vs. responses to two distinct faces. For an in-depth review, see (Schweinberger and Neumann, 2016). Previous studies (Amihai et al., 2011; Walther et al., 2013) have demonstrated that the N170 component is influenced when preceded by another face, regardless of whether the sequentially presented faces represent the same individual or different individuals. The present study provides further insight by showing that the categorical face adaptation effect accumulates over the duration of a session, leading to a gradual decline in N170 amplitude throughout the experimental session, as depicted in Figures 2E, 3E. Even when upright and inverted face stimuli were interleaved and separately analyzed, the apparent “neural adaptation effect” remained consistent across sessions. It’s noteworthy that this phenomenon has also been observed in data collected from the occipital lobe of rhesus macaque monkeys using non-face stimuli (Brunet et al., 2014). Therefore, researchers utilizing block designs to compare experimental conditions should consider this effect to ensure the reliability and validity of their interpretations.

### 4.6. Limitations of the study

It is essential to acknowledge certain limitations in our study. The homogeneity of our participant pool, primarily consisting of college students identifying as Hispanic/Latine, may influence our findings. Furthermore, the gender distribution in the sample was skewed toward females, suggesting the need for a more balanced participant pool in future investigations.

## Data availability statement

The raw data supporting the conclusions of this article will be made available by the authors, without undue reservation.

## Ethics statement

The studies involving humans were approved by Institutional Review Board of the California State University San Bernardino. The studies were conducted in accordance with the local legislation and institutional requirements. The participants provided their written informed consent to participate in this study.

## Author contributions

NB: Conceptualization, Formal analysis, Funding acquisition, Investigation, Methodology, Project administration, Resources, Supervision, Visualization, Writing – original draft, Writing – review & editing.
