# Supplementary material for: Face processing and early event-related potentials: replications and novel findings
Source: Front Hum Neurosci. 2023 Oct 25;17:1268972. doi: 10.3389/fnhum.2023.1268972 (PMC10634455; doi:10.3389/fnhum.2023.1268972)
Supplement: Supplementary file 1 [file Data_Sheet_1.PDF]

Supplementary Materials

Tables S1 to S5 presented below showcase the results of 3-way ANOVA analyses applied to each of the ERP components: P100, N170, and P200. These analyses were conducted on data obtained from both Experiment 1 (S1 and S2) and Experiment 2 (S3 to S5). Since each session comprised 416 trials, adopting a 4 or 5-way ANOVA would have necessitated averaging across either 26 or 13 trials, respectively. Such averaging could have potentially resulted in unstable ERP waveforms. Therefore, the approach of employing multiple 3-way ANOVAs, which only required averaging across 52 trials, was chosen. Significant findings are highlighted in yellow ($p < 0.05$).

Table S1: EXPERIMENT 1 (AGE, EMOTION, and MASK)

AGE / EMOTION/ MASK	P100				N170				P200			
	Sum Sq.	Mean Sq.	F	Prob →F	Sum Sq.	Mean Sq.	F	Prob →F	Sum Sq.	Mean Sq.	F	Prob →F
age	0.51	0.51	0.14	0.71	13.17	13.17	1.01	0.32	0.22	0.22	0.05	0.83
emotion	0.73	0.73	0.21	0.65	0.51	0.51	0.04	0.84	0.11	0.11	0.02	0.88
mask	0.19	0.19	0.05	0.82	119.75	119.7	9.2	0.003	46.03	46.03	10.16	0.002
age*emotion	0.29	0.29	0.08	0.78	0.11	0.11	0.01	0.93	1.83	1.83	0.40	0.53
age*mask	1.32	1.32	0.37	0.54	2.10	2.10	0.16	0.69	4.41	4.41	0.97	0.33
emotion*mask	0.75	0.75	0.21	0.65	0.00	0.00	0.00	1.00	0.00	0.00	0.00	0.99

Table S2: EXPERIMENT 1 (GENDER, EMOTION, and MASK)

GENDER/RACE/ MASK	P100				N170				P200			
	Sum Sq.	Mean Sq.	F	Prob →F	Sum Sq.	Mean Sq.	F	Prob →F	Sum Sq.	Mean Sq.	F	Prob →F
gender	0.33	0.33	0.10	0.76	18.44	18.44	1.44	0.23	10.83	10.83	2.41	0.12
race	1.94	1.94	0.58	0.45	0.02	0.02	0.00	0.97	2.55	2.55	0.57	0.45
mask	0.27	0.27	0.08	0.78	118.18	118.18	9.24	0.003	48.87	48.87	10.88	0.001
gender*race	1.37	1.37	0.41	0.52	0.07	0.08	0.01	0.94	1.60	1.60	0.36	0.55
gender*mask	2.03	2.03	0.60	0.44	0.00	0.00	0.00	0.99	1.09	1.09	0.24	0.62
race*mask	0.56	0.56	0.17	0.68	1.88	1.88	0.15	0.70	1.16	1.16	0.26	0.61

Table S3: EXPERIMENT 2 (AGE, STIMULUS ORIENTATION, and MASK)

AGE/ORIENTATION? MASK	P100				N170				P200			
	Sum Sq.	Mean Sq.	F	Prob →F	Sum Sq.	Mean Sq.	F	Prob →F	Sum Sq.	Mean Sq.	F	Prob →F
age	0.58	0.58	0.09	0.77	12.32	12.32	0.77	0.38	1.11	1.11	0.17	0.68
orientation	21.30	21.30	3.16	0.08	82.38	82.38	5.18	0.025	12.60	12.60	1.97	0.16
mask	1.19	1.19	0.18	0.68	144.27	144.27	9.07	0.003	76.33	76.33	11.91	0.001
age*orientation	3.00	2.99	0.44	0.51	8.00	8.00	0.50	0.48	0.01	0.01	0.00	0.96
age*mask	0.80	0.80	0.12	0.73	0.04	0.04	0.00	0.96	4.23	4.23	0.66	0.42
orientation*mask	2.24	2.24	0.33	0.57	69.61	69.61	4.37	0.039	22.82	22.82	3.56	0.06

Table S4: EXPERIMENT 2 (GENDER, STIMULUS ORIENTATION, and MASK)

GENDER/ ORIENTATION/MASK	P100				N170				P200			
	Sum Sq.	Mean Sq.	F	Prob →F	Sum Sq.	Mean Sq.	F	Prob →F	Sum Sq.	Mean Sq.	F	Prob →F
gender	1.39	1.39	0.20	0.65	14.22	14.22	0.88	0.35	1.68	1.68	0.27	0.61
orientation	21.27	21.27	3.14	0.08	81.33	81.33	5.06	0.027	8.99	8.99	1.42	0.24
mask	0.90	0.90	0.13	0.72	149.03	149.03	9.27	0.003	74.23	74.23	11.75	0.001
gender*orientation	0.01	0.01	0.00	0.98	0.28	0.28	0.02	0.90	2.15	2.15	0.34	0.56
gender*mask	2.26	2.25	0.33	0.57	14.15	14.15	0.88	0.35	0.51	0.51	0.08	0.78
orientation*mask	2.44	2.44	0.36	0.55	65.31	65.31	4.06	0.046	26.59	26.59	4.21	0.043

Table S4: EXPERIMENT 2 (RACE, STIMULUS ORIENTATION, and MASK)

RACE/ORIENTATION/ MASK	P100				N170				P200			
	Sum Sq.	Mean Sq.	F	Prob →F	Sum Sq.	Mean Sq.	F	Prob →F	Sum Sq.	Mean Sq.	F	Prob →F
race	0.96	0.96	0.14	0.71	0.24	0.24	0.01	0.90	1.74	1.74	0.27	0.61
orientation	21.31	21.31	3.05	0.08	80.15	80.15	4.91	0.029	13.84	13.84	2.14	0.15
mask	0.85	0.85	0.12	0.73	148.53	148.53	9.09	0.003	69.44	69.44	10.72	0.001
race*orientation	1.30	1.30	0.19	0.67	0.39	0.39	0.02	0.88	0.22	0.22	0.03	0.85
race*mask	0.53	0.53	0.08	0.78	1.49	1.49	0.09	0.76	0.07	0.07	0.01	0.92
orientation*mask	2.11	2.11	0.30	0.58	69.95	69.95	4.28	0.041	27.02	27.02	4.17	0.043

Figure S1

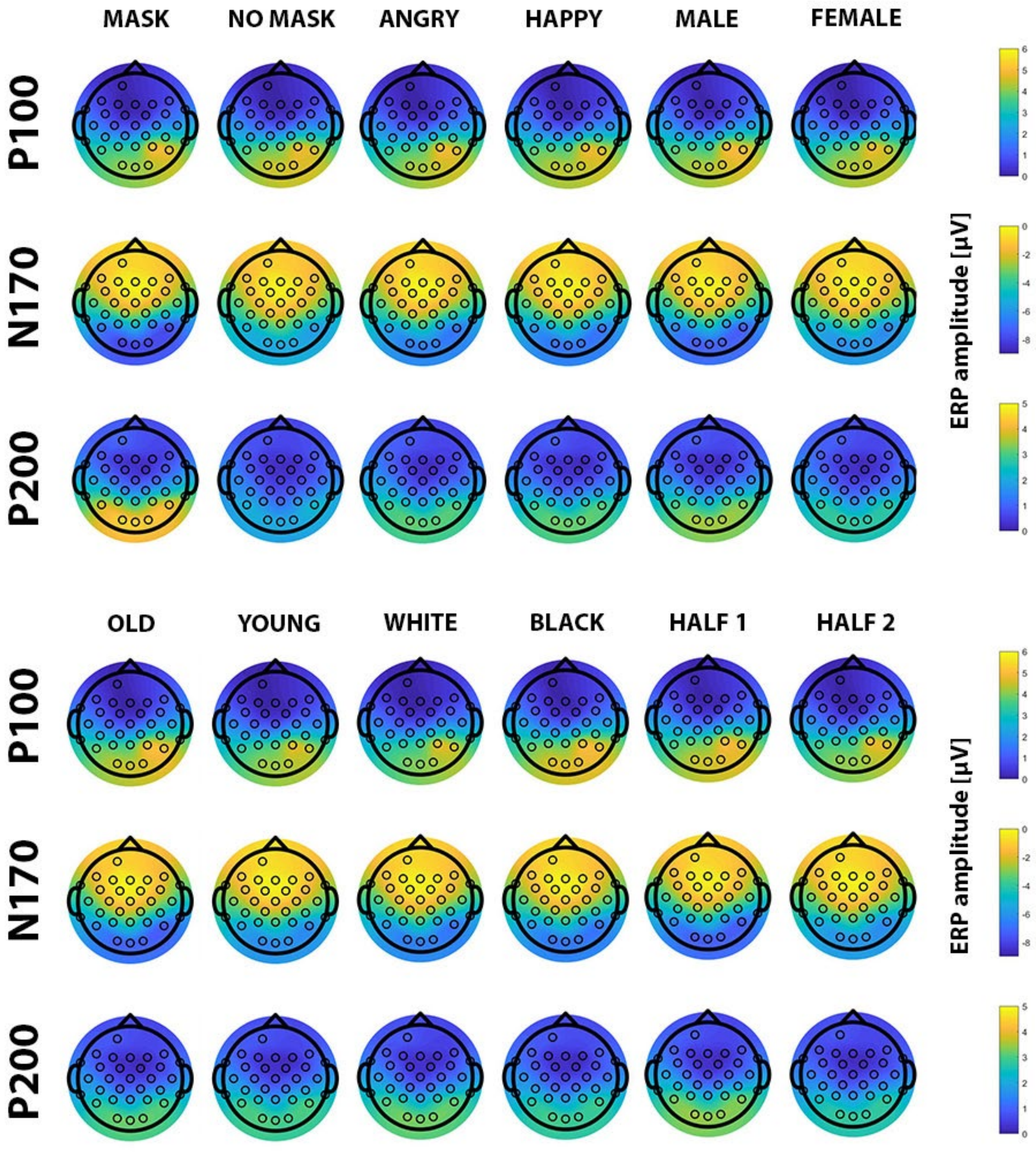

<u>Figure S1</u>: *Topographical maps illustrate the data collected in Experiment 1. Each map represents the average data from participants, calculated based on 50% of the trials. These trials are categorized based on either five pairs of binary dimensions or simply the first and second halves. This categorization is applied to each of the three ERP components: P100, N170, and P200. Although distinct scales are employed for each component, uniformity is maintained across all conditions. Detailed explanations regarding the selection of ERP maxima can be found in the method section.*

Figure S2

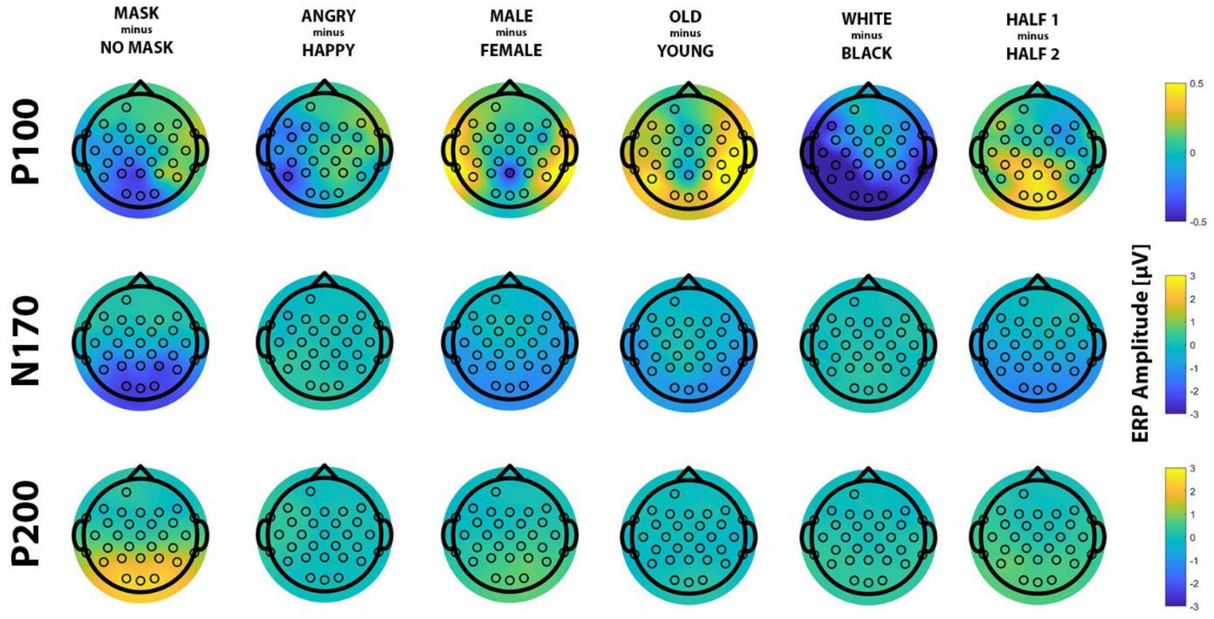

<u>Figure S2</u>: *Following the same approach as in Figure S1. However, the data in this figure is obtained by subtracting each pair of the five binary dimensions from each other. This subtraction is also applied to the data computed separately for the first and second 50% of the trials. Additionally, distinct scales are employed for each ERP component, with uniformity maintained across different conditions.*

Figure S3

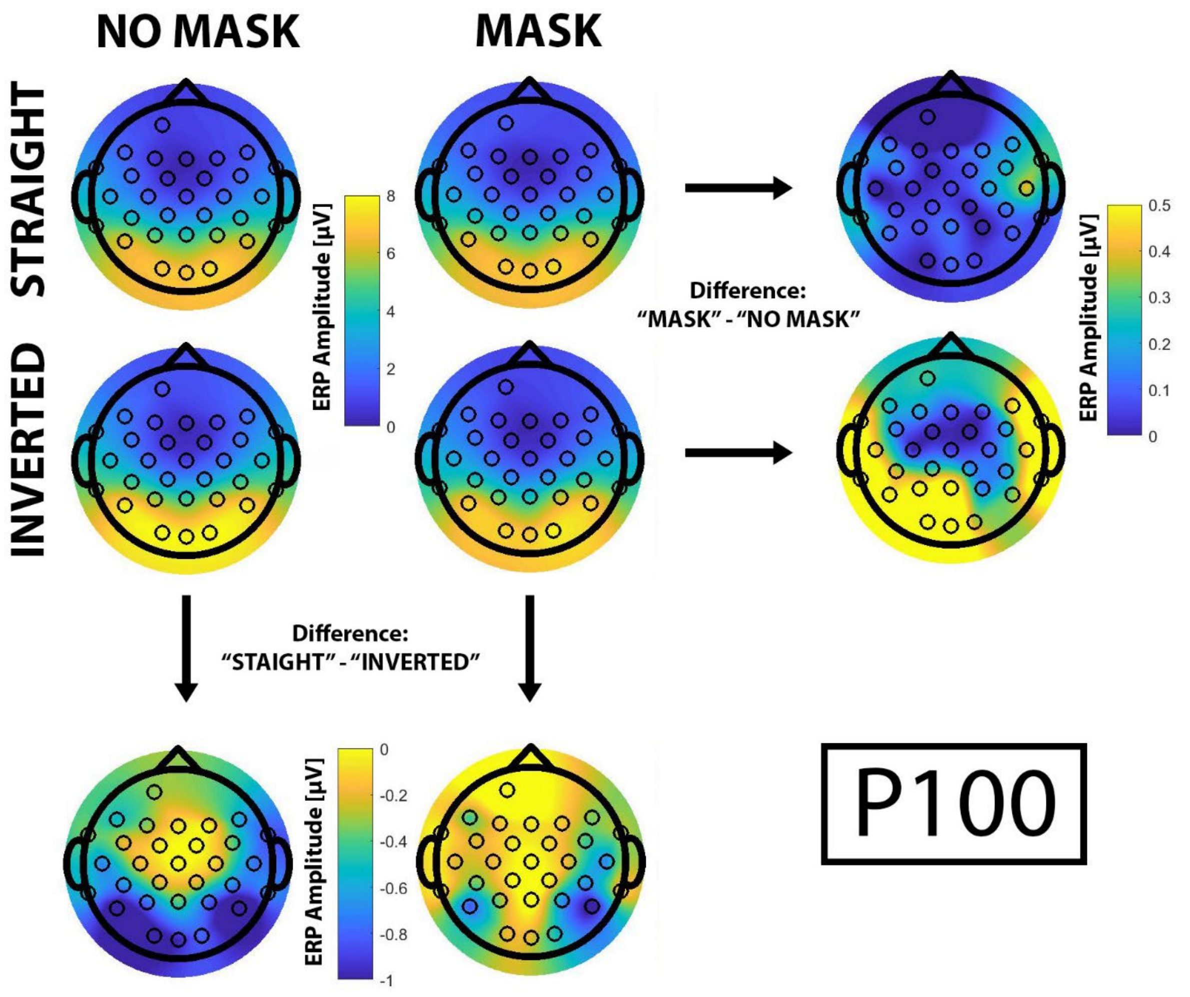

Figure S3: *Topographical maps depicting P100 ERP components extracted from Experiment 2 data are presented. The four maps in the upper-left corner represent participant-averaged data, each based on 25% of the trials. These trials involved faces that were either masked or unmasked and displayed in either an upright or inverted orientation, all using the same scale. Results obtained by subtracting the two mask conditions and the two orientation conditions are respectively displayed to the right and below. Distinct scales were thoughtfully applied in each case to facilitate an understanding of the P100 amplitude distribution. Comprehensive explanations regarding the selection of P100 maxima can be found in the method section.*

Figure S4

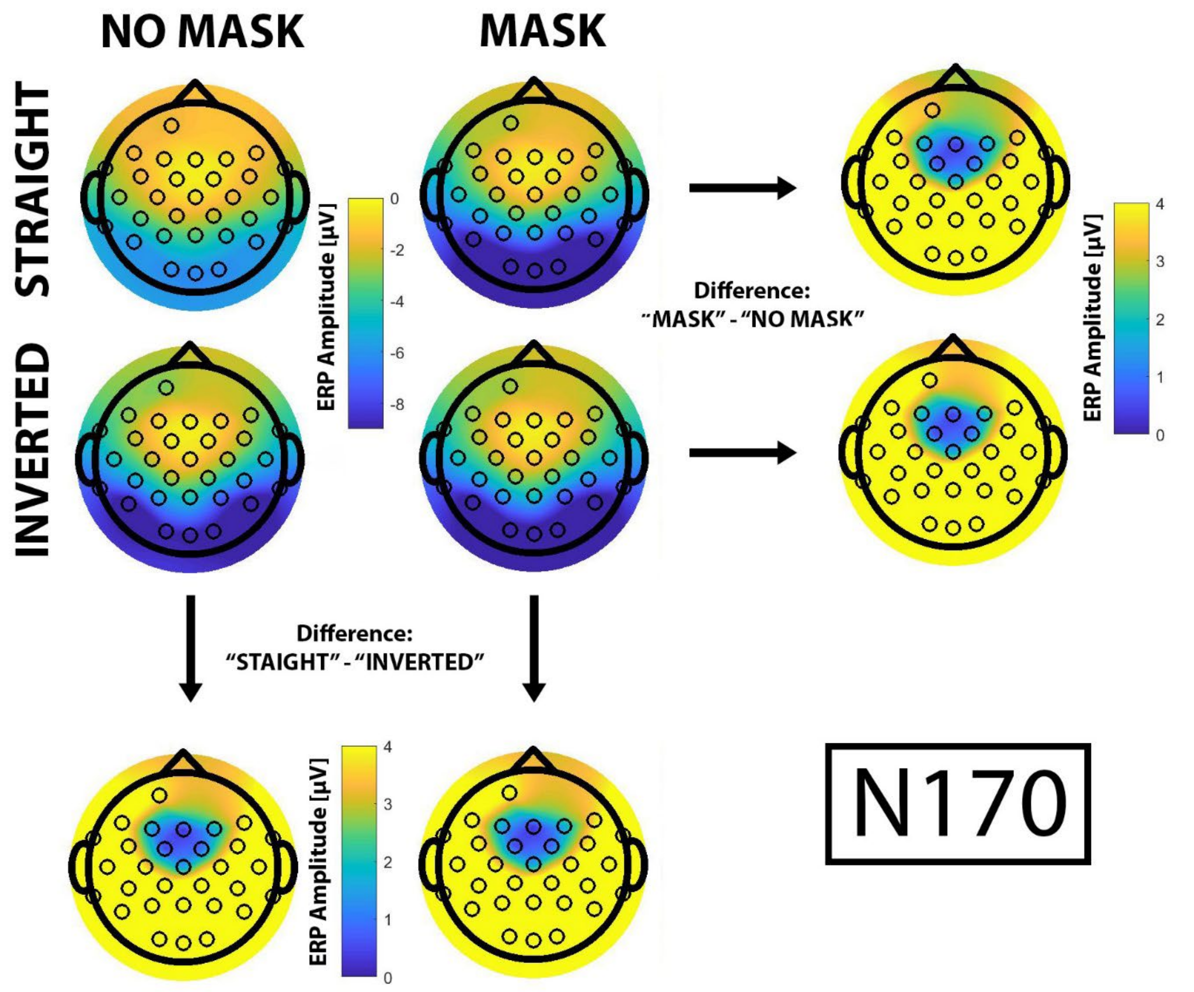

<u>Figure S4</u>: Similar to Figure S3, but with a focus on the N170 ERP component instead of the P100 component.

Figure S5

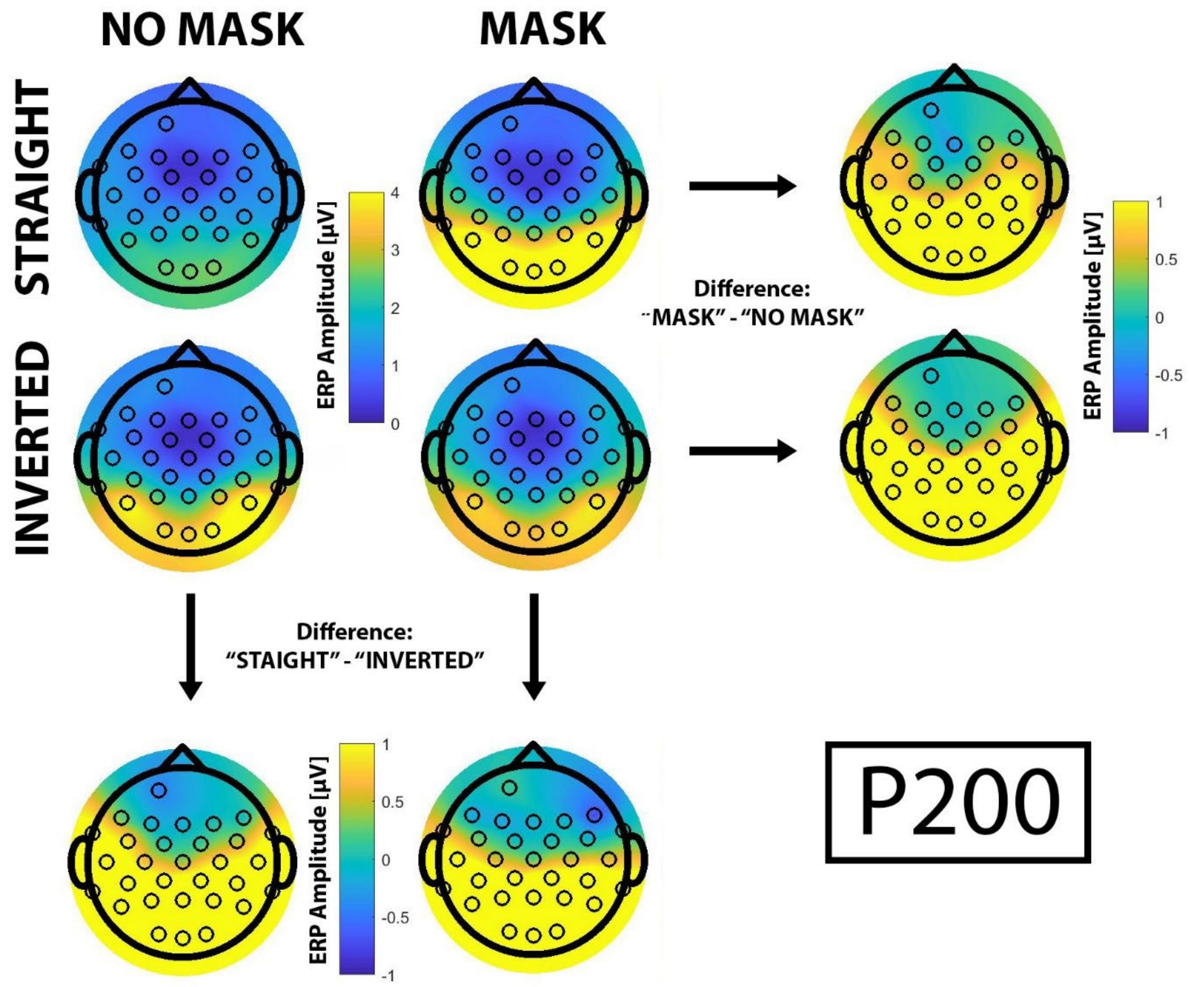

<u>Figure S5</u>: Similar to Figure S3, but with a focus on the P200 ERP component instead of the P100 component.
